# Shape-Memory Polymers Based on Carbon Nanotube Composites

**DOI:** 10.3390/mi15060748

**Published:** 2024-06-01

**Authors:** Mariana Martins da Silva, Mariana Paiva Proença, José António Covas, Maria C. Paiva

**Affiliations:** 1 Institute for Polymers and Composites, University of Minho, Campus of Azurém, 4800-058 Guimarães, Portugal; mmsilva@dep.uminho.pt (M.M.d.S.); jcovas@dep.uminho.pt (J.A.C.); 2 ISOM and Departamento de Electrónica Física, Universidad Politécnica de Madrid, Ava. Complutense 30, E-28040 Madrid, Spain; mariana.proenca@upm.es

**Keywords:** shape-memory polymers, carbon nanotubes, soft actuators, nanocomposites

## Abstract

For the past two decades, researchers have been exploring the potential benefits of combining shape-memory polymers (SMP) with carbon nanotubes (CNT). By incorporating CNT as reinforcement in SMP, they have aimed to enhance the mechanical properties and improve shape fixity. However, the remarkable intrinsic properties of CNT have also opened up new paths for actuation mechanisms, including electro- and photo-thermal responses. This opens up possibilities for developing soft actuators that could lead to technological advancements in areas such as tissue engineering and soft robotics. SMP/CNT composites offer numerous advantages, including fast actuation, remote control, performance in challenging environments, complex shape deformations, and multifunctionality. This review provides an in-depth overview of the research conducted over the past few years on the production of SMP/CNT composites with both thermoset and thermoplastic matrices, with a focus on the unique contributions of CNT to the nanocomposite’s response to external stimuli.

## 1. Introduction

Being mainly designed to mimic biological systems, shape-memory polymers (SMP) rely on the presence of soft and hard segments within the polymeric matrix to retain and change shape. Physical interactions in thermoplastic polymers, or chemical crosslinks in thermoset polymers, help fix a particular shape, whereas chain network mobility allows their movement, enabling SMP to perform a series of deformations, such as expansion, bending, and torsion.

SMP respond to a stimulus [1,2,3], the most common being a temperature change, either by direct or indirect heat sources. In this case, the motion of the polymer chains is key, and thus, the transition temperatures ($T_{trans}$)—either glass transition temperature ($T_{g}$) or melting temperature ($T_{m}$)—play a fundamental role in shape deformation, enabled by storing and releasing the energy of polymer chain interactions.

**Figure 1:**
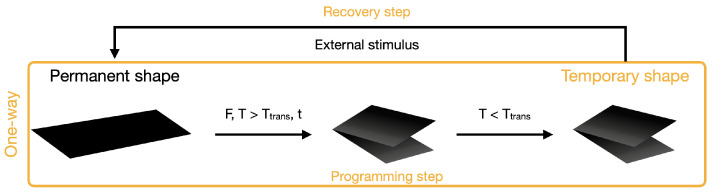
Schematic illustration of a one-way shape-memory deformation mechanism. In a one-way type of deformation, a programming step, with an application of mechanical stress (F), temperature (T), and time (t), is required to have a temporary shape that will then be subject to an external stimulus to deform.

The great majority of the reported SMP need a programming step before each deformation cycle occurs [4,5]. A one-way shape-memory effect is illustrated in Figure 1: during the programming step, a shape is given by heating the polymer above a $T_{trans}$, holding it mechanically in the desired form, and cooling it down to constrain chain mobility, retaining the temporary shape. When a certain stimulus is applied, the mobility of the polymer chains is affected, a rubbery state is attained, and the polymer relaxes and returns to its initial permanent shape. One-way SMP remember only one shape, the permanent one.

Two-way and more shape-memory effects are based on the same mechanism of actuation, i.e., the polymer undergoes a programming step before every actuation; however, it now allows programming two or more temporary shapes. This effect can be achieved for polymer systems with more than two transition temperatures, $T_{trans\;1}$ and $T_{trans\;2}$. Temporary shape 1 will be associated with $T_{trans\;1}$, temporary shape 2 with $T_{trans\;2}$, and so on. Applying an external stimulus will allow the polymer chains to return to their initial form, moving from temporary shape 2 to 1 and back to the permanent shape.

**Figure 2:**
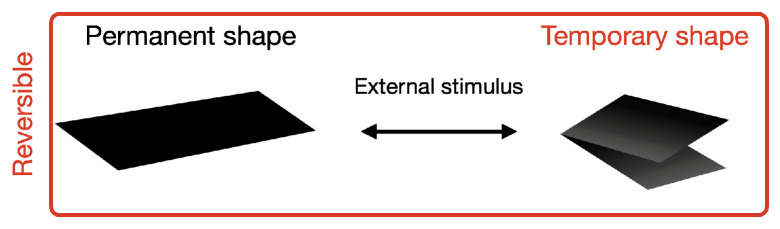
Schematic illustration of a reversible shape-memory deformation mechanism.

In a reversible SMP, there is no need for a programming step before every actuation (Figure 2). An external stimulus applied to an initial (permanent) shape alters the polymer chain mobility, endowing deformation to a distinct (temporary) shape and retaining the same until the stimulus is cut off. The removal of the stimulus causes the polymer chains to relax, recovering their permanent shape. For some systems, it is possible to provide a stimulus at different levels and obtain more than one temporary shape [6,7].

Besides direct heat, other stimuli can induce actuation, allowing remotely actuated systems and faster response times [4,5,8,9,10]. The different stimuli and actuation mechanisms reported in the literature, together with their advantages and disadvantages, are summarized in Table 1.

Adding fillers to a polymer matrix allows the exploration of mechanisms of actuation otherwise not possible. Among popular reinforcements for SMP, carbon-based fillers, such as carbon fibers (CFs), carbon black (CB), carbon nanotubes (CNT), and graphene (Gr), are popular for electrically responsive SMP, Fe_3_O_4_ and Ni for magnetically responsive SMP, nanocellulose for chemically activated SMP, and Au, CNT, or graphene oxide (rGO) for light-responsive SMP [11,12,13,14,15].

This review aims to provide an overview of thermoplastic and thermoset SMP filled with CNT, focusing on the role of CNT in the shape-memory effect (SME). The fabrication of SMP is discussed briefly in the first section. The actuation mechanisms of SMP with CNT are further detailed, as well as the multifunctionality of CNT-filled SMP.

## 2. Processing SMP with Carbon Nanotubes

The movement of SMP relies on the nature of the polymer chains, their molecular weight, the phase domains separating hard and soft segments, and the chemical crosslinking degree [11]. To enable permanent and temporary shapes, SMP are often designed with two distinct phases, achieved mainly through the assembly of block co-polymers and blends [1]. One of the phases is meant to fix the permanent shape, formed with harder polymeric segments, and the other with soft chains, allowing sufficient mobility for the SMP to return to its permanent shape.

Adequate processing routes should be selected to maintain the integrity of the CNT in composites, while ensuring a homogeneous dispersion tailored to the target application. The concept of 4D printing, mentioned vastly in the literature for the past 10 years, has been used to define 3D-printed structures that, when subjected to a certain external stimulus, are capable of morphing [14,16,17,18,19,20,21].

Much has been reported concerning carbon-filled polymer composites, their potential applications, and their processing routes. The following sections present a summary of the properties of CNT, and the effect of the addition of CNT to thermosetting and thermoplastic polymer matrices for the assembly of SMP.

### 2.1. Properties of Carbon Nanotubes

CNT are cylindrical graphene (Gr) structures of a few nanometers in diameter and lengths that can reach several hundreds of microns, formed by a hexagonal lattice of strongly bonded $sp^{2}$ hybridized carbon atoms [22]. CNT can be either single-walled (SWCNT), consisting of one wall tube formed by a rolled Gr sheet, or multi-walled (MWCNT), composed of several concentric layers of rolled Gr sheets. SWCNT tend to strongly attach to each other through van der Waals forces, packing into tight bundles, as they present fewer defects than MWCNT. Thus, SWCNT present higher electrical conductivity, and better mechanical and thermal properties overall compared to MWCNT.

CNT are stable in air up to near 600 °C [23]. The high thermal conductivity (TC) in carbon materials is dominated by atomic vibrations and phonons, potentially enhancing the TC of polymer composites at low CNT loading. However, experimental results for polymer composites show modest results in TC enhancement. This relates to (i) high thermal resistance between individual CNT [24] leading to a TC of ∼30 W·m^−1^·K^−1^ for SWCNT buckypapers [25], while theoretical experiments indicate a thermal conductivity of 6000 W·m^−1^·K^−1^ for a single SWCNT [26], and (ii) the limitations of phonons in traveling through the matrix [27]. Overall, the TC of CNT/polymer composites depends on the CNT content, aspect ratio, dispersion, and interfacial interaction [28]. The coefficient of thermal expansion (CTE) of CNT is near identical to the isotropic CTE of C–C bonds (around −1.5 ppm·K^−1^), contrasting with a positive CTE for polymers in the range of 20–200 ppm·K^−1^ (Table 2). Thus, actuation based on thermal expansion depends on a large CTE of the polymeric matrix. A polymer with weaker bonds presents a higher CTE, so thermoplastic matrices are expected to present a higher CTE, while elastomers present negative values when stretched above $T_{g}$ [29,30].

The absorption of electromagnetic (EM) radiation is observed in a wide range of wavelengths for CNT. Emissivities of ∼0.98 for wavelengths ranging from 5 to 12 μm and reflectance of ∼0.02 from far ultra-violet (UV, 200 nm) to far infra-red (IR, 200 μm) have been reported for vertically aligned SWCNT forests [34]. CNT act as electron carriers, transforming absorbed energy from photons into heat [15].

CNT/polymer composites may be electrically conductive, considering the high electrical conductivity of CNT [35] (Table 2). The polymer/CNT composite’s overall conductivity is governed by a percolation threshold observed when the electrically insulating composite rapidly increases electrical conductivity at a critical CNT concentration. At the threshold, a conductive network is formed throughout the composite volume. Due to the high aspect ratio and nano size of the CNT, electrical conductivity may be achieved at CNT loadings as low as 0.1 wt.% [36,37].

The efficient mechanical strengthening of a polymer matrix occurs when CNT are individually and homogeneously dispersed within the polymeric matrix, leading to efficient load transfer and an enhanced Young’s modulus and tensile strength of the composites [38].

The main limitation of CNT as fillers in polymers is their entangled form and tendency to form agglomerates, which are stabilized by van der Waals forces, and weak interfacial adhesion with polymers. Purification, surface chemical modifications, alignment, and pre-dispersion of CNT, alongside the adjustment of the processing techniques, are often proposed to maximize the dispersion of CNT, disentanglement, and improve interface interactions with the matrix [23,27,28,37,39,40]. The covalent functionalization of CNT [41,42,43,44] is one of the strategies to improve surface adhesion between the filler and polymer, but it may affect the intrinsic properties of the CNT. Non-covalent modifications, such as the use of plasticizers [6,45,46,47,48,49,50] and pre-dispersions of CNT [40,51,52], may lead to superior polymer/CNT interface interactions and improved processability.

### 2.2. Thermoset Composites with Carbon Nanotubes

Thermoset polymers usually display a high modulus and high $T_{g}$ with low deformability. As SMP, this class of polymers is able to operate at high temperatures and stress requirements, with a lower strain ability compared to thermoplastics [53]. Epoxy resin (ER) and thermoset polyurethane (PU) are the most used polymers for SMP composites with CNT. Thermoset SMP can also be prepared by adding linear monomers or thermoplastic segments or by chemically crosslinking functional polymer chains, such as PU/styrene systems [53].

The low viscosity of the monomers/pre-polymer that are used to obtain a thermoset is an advantage for dispersing CNT. A thermoset can be compounded with CNT either by milling (three-roll mill and ball mill), sonication, high shear mixing, and the impregnation of resin into the CNT. A step of curing follows the compounding to harden the polymer. The dispersion of CNT in the polymer drastically increases the viscosity of the composite; hence, voids are commonly formed during mixing and curing.

Over the past years, additive manufacturing technologies have been explored to process thermoset SMP. The digital light process (DLP) and Stereolithography (SLA) 3D printing techniques imply the use of liquid resins and composites. As the remaining 3D printing techniques, these are based on computer-aided design (CAD) models, sliced into horizontal layers, which are subsequently printed on top of each other [21,54]. Thermoset consolidation is frequently achieved by photopolymerization, thus requiring a photocurable resin. However, CNT absorb a part of the UV radiation, thus limiting the curing depth of the resin [55].

Remarkable work for SMP has been developed with liquid crystal elastomers (LCEs) and vitrimers [56], the first due to their low crosslinking degree, whilst the second can undergo thermally activated bond exchange reactions. These sub-sets of thermoset polymers will not be discussed in this review, yet reviews regarding composites made with LCE or vitrimers with carbon fillers may be found in the literature [56,57,58,59].

### 2.3. Thermoplastic Composites with Carbon Nanotubes

The most common processing routes to obtain CNT thermoplastic nanocomposites are solvent casting, in situ polymerization, and melt mixing [5,37,39]. The dispersion of CNT is facilitated in low viscosity media such as solvents or liquid monomers. Direct dispersion in the polymer melt, albeit more complex due to its high viscosity, is better suited for scaling up to industrial production, avoiding the management of waste solvents. The effective dispersion of CNT in a polymer melt requires the optimization of the processing conditions, namely temperature, the application of high shear stress, and the duration of the mixing action [44,60]. Strategies such as layering polymers have also been used to produce SMP with enhanced control of the micro- and nano-domains, based on continuous layers, in opposition to the co-continuous phases of blends and block copolymers [3,61,62,63].

Composite filaments with a controlled diameter, obtained by melt extrusion techniques, are the base material for fused deposition modeling (FDM), a 3D printing technique. The filament is heated to a melt in the printer nozzle, and a thin thread is deposited in successive horizontal layers according to a CAD design [21,54]. A major limitation of the technique is related to the limited adhesion between consecutive composite layers, affecting the overall mechanical properties, surface quality, shape fixity, and recovery [21].

Thermoplastic polyurethane (TPU), poly(ethylene-co-vinyl acetate) (EVA), poly(ϵ-caprolactone) (PCL), and poly(lactic) acid (PLA) are the most commonly used thermoplastic polymers to assemble SMP with CNT [29,64,65,66]. Polymer blends are widely used to design SMP. Melt-blending polymers, or polymer composites, may lead to the development of co-continuous phases with different morphologies, enabling the control of hard and soft segments and phase differences, as well as the localization of nanoparticles in the polymer phases. SMP may be prepared with CNT selectively localized in a specific polymer in immiscible polymer blends [67]. The selective location of CNT in blends can be estimated thermodynamically through a wetting coefficient that relates the interfacial energy between the CNT and each polymer in the blend, as well as the interfacial energy of the two polymers in contact, as described by Sumita et al. [68].

**Figure 3:**
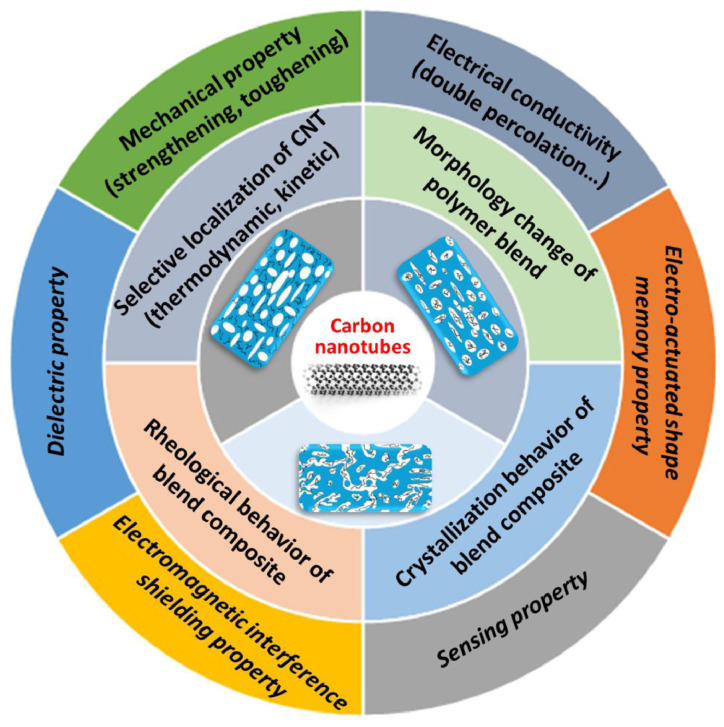
Summary of the effect of the selective localization of CNT on the properties of the corresponding polymer blends. The inset pictures represent CNT selectively located in the dispersed domains or in the continuous component of binary immiscible blends. Reprinted with permission from Qi et al. [67]. Copyright 2021, Elsevier.

The addition of CNT may change the morphology of the phase domains in polymer blends. For example, in EVA/PCL blends (60:40), the CNT showed affinity towards the EVA phase; increasing the concentration of CNT led to the formation of smaller PCL phase domains, and the crystallization temperature of EVA was slightly increased [69]. For TPU/Acrylonitrile butadiene styrene (ABS) blends, the addition of MWCNT, with an affinity towards TPU reduced the size of ABS droplets in a sea island morphology [70]. Often used for SMP, PLA is rather brittle, and hence, blending with other polymers, such as PCL [71,72] and TPU [50,73,74,75,76], is reported. The blend morphology may change by varying the polymer ratio and CNT concentration. PLA/TPU (50:50) blends without CNT present a co-continuous morphology, while the addition of CNT leads to a sea island morphology, with PLA phase domains decreasing in size for higher amounts of CNT [77], whereas a PLA/TPU (10:90) blend presents a co-continuous structure [73]. Figure 3 illustrates how the blend morphology and selective localization of CNT influences the functional properties of composite blends [67].

## 3. Thermal Actuation of SMP with CNT

### 3.1. Direct Heating

**Figure 4:**
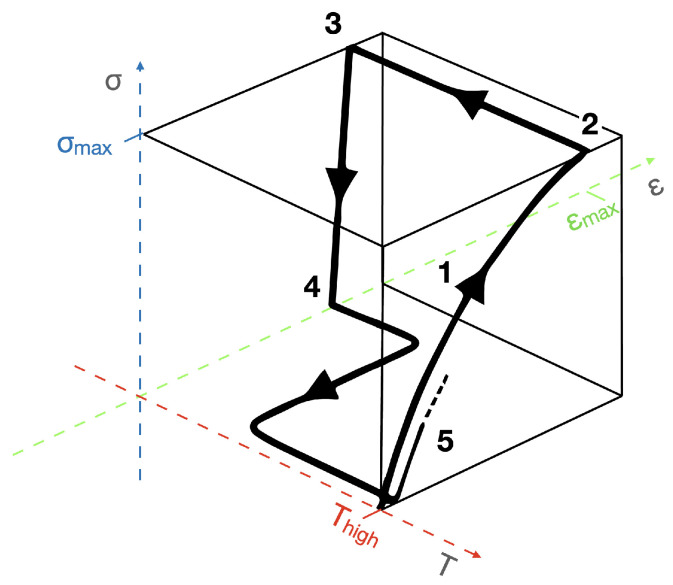
Schematic representation of the four steps of the cyclic thermomechanical tests. Adapted with permission from Lendlein and Kelch [29]. Copyright 2002, John Wiley and Sons.

In 2002, Lendlein and Kelch [29] proposed the quantification of the response of a thermally responsive SMP (t-SMP) by means of cyclic thermomechanical tests, quantifying a shape fixity ratio ($R_{f}$) and a shape recovery ratio ($R_{r}$). The cyclic thermomechanical test program is represented in Figure 4. In the first step (1), the sample is stretched to a maximum strain ($\epsilon_{max}$), at a temperature higher than $T_{trans}$ ($T_{high}$). Then, the sample is cooled down while the tensile stress is kept constant at $\sigma_{max}$ (step 2). The clamp distance is then reduced until a stress-free condition is attained (σ=0MPa, step 3). The ratio of the tensile strain after unloading ($\epsilon_{u}$) and the maximum strain ($\epsilon_{max}$) (Equation (1)) (at the *n*th cycle) quantifies the ability of the t-SMP to retain the mechanical deformation applied during the programming step, the shape fixity ratio ($R_{f}$).

(1) $$R_{f}\;\left(n\right)=\frac{\epsilon_{u}\;\left(n\right)}{\epsilon_{max}}$$

When the t-SMP returns to its permanent shape, the strain applied during programming is recovered as a result of heat application, as depicted in Figure 4, step 4. The shape recovery ratio ($R_{r}$) is a measure of how well the permanent shape was memorized and can be calculated according to Equation (2):

(2) $$R_{r}\;\left(n\right)=\frac{\epsilon_{max}\;\epsilon_{p}\;\left(n\right)}{\epsilon_{max}-\epsilon_{p}\;\left(n-1\right)}$$

where $\epsilon_{p}$ is the recovered strain of a sample, in this case in two successive cycles *n* and n−1.

Bending tests can also characterize an SME. The sample is bent at a given angle ($\theta_{i}$) and cooled below $T_{trans}$. Subsequent release of the stress and heating of the sample above $T_{trans}$ will cause a deformation, as the angle will change as a function of time. $R_{r}$ can then be calculated through Equation (3) [78]:

(3) $$R_{r}=\frac{\left(\theta_{i}-\theta_{f}\right)}{\theta_{i}},$$

$\theta_{f}$ being the angle at a given time.

The presence of CNT in the composite often decreases $R_{r}$ and increases $R_{f}$ compared to the neat t-SMP. The addition of CNT increases the storage modulus and interferes with the motion of polymeric chains. The stronger the interaction between the CNT and polymer, and the higher the concentration of CNT (up to a critical value), the more likely it is that the thermal stability of a nanocomposite will increase. Typically, for a composite with a strong CNT/polymer interface, $T_{trans}$ will increase relative to the neat polymer. Few studies of SMP/CNT analyzed the thermal conductivity (TC) of composites; this is an important property of thermally activated (directly or not) SMP since an increase in TC will enhance the heat transfer process and potentially decrease the actuation time. The TC of a t-SMP was studied by Yin et al. [79]; CNT were added to accelerate the heat transfer and phase change by increasing the melting/crystallization rate; a difference of ∼0.6 W·m^−1^·K^−1^ was found between the neat poly(acrylamide-octadecyl acrylate) (P(AM-co-OA)) and the composites. The TC of TPU/CNT reported by Vishwakarma et al. [80] was found to be similar to neat TPU at 25 °C, while at 50 °C and 75 °C, it decreased for neat TPU in comparison. Xia et al. [81] reported on a small increase of the TC for Trans-1,4-polyisoprene (TPI)/low-density polyethylene (LDPE) filled with CNT. Kang et al. [82] observed a threshold increase of the TC for TPU/hybrid SWCNT (hSWCNT) composites compared to neat TPU (0.33 W·m^−1^·K^−1^); composites prepared with 2 wt.% reduced graphene oxide (rGO) and SWCNT also revealed differences in TC depending on the processing routes followed. Only a few studies reported on the TC of SMP actuated through indirect heat stimuli [73,83,84,85,86], most studies referring to TPU matrices with MWCNT.

Most of the designed t-SMP have one or more-way shape-memory effect, thus requiring programming at a temperature higher than $T_{trans}$, with no reversibility, only re-usability. Controlling the temperature is required to operate t-SMP: an oven, a hot plate, or a water bath are the most common heat sources to induce shape deformations.

A list of research works reported in the literature concerning thermoset/CNT t-SMP nanocomposites is presented in Table 3, indicating the filler load, polymer matrix, processing method, recovery, and fixity rates, as well as the $T_{trans}$ selected for mobility and the temperature at which the experiment was carried out.

As mentioned in Section 2.2, ER and PU are among the most used thermoset polymer matrices to process nanocomposites through mechanical stirring, followed by curing. Both pristine and functionalized MWCNT (fMWCNT) are commonly used as fillers, contributing to an increase of the mechanical properties compared to neat resin, increasing $R_{f}$. The recovery of the temporary shape typically takes less than 90 s with temperatures of actuation between 40 °C (PU resin with 2 wt.% of MWCNT [99]) and 190 °C (cyanate ester (CE) resin with 2 wt.% fMWCNT [101]). An $R_{r}$ of 96% with a recovery time of 5 s was reported by Markad and Lal [89] for ER filled with 0.4 to 0.6 wt.% of -COOH and -NH_2_ fMWCNT, compared to a recovery time of 9 s for neat ER. The authors have observed a decline in the mechanical properties, with increased recovery time at 0.8 wt.% fMWCNT, due to the agglomeration of CNT in the matrix.

Tang et al. [91] studied the shape-memory performance of ER/MWCNT composites with different diameters, namely 8–15 nm (50 μm length) and 30–50 nm (10–20 μm length), observing that smaller CNT diameters were more effective at increasing the composite’s tensile strength and elongation at break. For lower diameter MWCNT at 1 wt.%, it was possible to achieve a faster recovery speed with a high $R_{r}$; in contrast, MWCNT with a larger diameter contributed to enhancing the heat resistance. Reversible plasticity shape-memory (RPSM) properties were reported by Abishera et al. [94] for an ER reinforced with MWCNT. For RPSM, the programming temperature was set below $T_{g}$; in their work, the programming temperature was set to ($T_{g}-15$ °C). It was observed that MWCNT increased the mechanical and RPSM properties, namely improved shape fixity, response temperature, and recovery speed.

**Figure 5:**
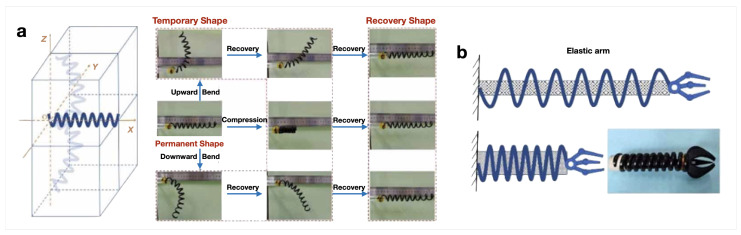
Spring actuator composed of CE with COOH-MWCNT/CFs: (**a**) shape recovery process of a spring in space and (**b**) spring elastic arm. Adapted with permission from Wang et al. [101]. Copyright 2022, IOP Publishing.

For a high actuation temperature and operation in space, Wang et al. [101] developed springs of CE resin with 2 wt.% of fMWCNT (COOH-MWCNT) and 2 wt.% of carbon fibers (CF) (Figure 5a). The spring was stimulated at 190 °C with the aim to be used as an elastic arm (Figure 5b), and the authors observed that the $R_{r}$ of the composites increased in comparison to neat CE.

Similar to thermosets, MWCNT and fMWCNT are frequently used fillers for t-SMP based on thermoplastics. Overall, the addition of fMWCNT aids in increasing $R_{f}$. Surface modifications of CNT may enhance the interface interaction between fillers and the matrix, reducing the negative effect of CNT on the $R_{r}$. Table 4 presents a comprehensive list of the works reporting t-SMP based on CNT composites with thermoplastic matrices. Recovery times are reported to be faster than for thermoset t-SMP, with the great majority being able to recover the permanent shape after 72 s. Actuation in 10 s is reported for bi-layers of TPU with hybrid Ag/MWCNT fillers [102] and polyaryletherketone (PAEK) with 10 wt.% of CNT [103]. As the most common matrix for t-SMP, TPU presents a negative $T_{g}$, which enables actuation at low temperatures, with the lowest actuation temperature reported at 10 °C for both MWCNT and fMWCNT filled TPU [104]. PAEK composites with up to 15 wt.% MWCNT can actuate at high temperatures, ∼181 °C [105]. The most commonly used composite preparation method is melt mixing, followed by compression molding, to produce the composite with the desired shape.

**Figure 6:**
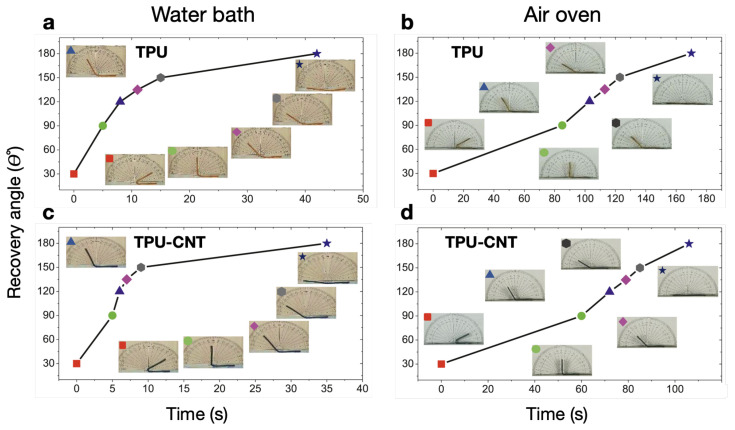
Progressing shape-memory recovery, as a function of time, of TPU, and TPU/CNT composites in a hot water bath (**a**,**c**) and a hot air oven (**b**,**d**). Adapted with permission from Vishwakarma et al. [80]. Copyright 2022, Elsevier.

In 2014, Koerner et al. [133] produced TPU/MWCNT composites, reporting that dispersed CNT alter the strain dependence of polymer crystallite formation, which, in turn, increased the strain set (fixity) and stored energy density. Yusrizal et al. [126] prepared a series of composites of fMWCNT (OH-MWCNT) and TPU with different contents of palm oil polyol (POP) in the soft segments, reporting that $R_{f}$ was 100% for neat TPU and composites with POP below 30 wt.%, above which neat TPU presented an $R_{f}$ up to 16% lower. The addition of fMWCNT loads in the range of 0.5–2 wt.% attenuated the negative effect on $R_{f}$ induced by POP. $R_{r}$ was kept at 100% for all compositions, and the recovery was faster for composites with fMWCNT compared to neat TPU, decreasing the recovery time with the increase in fMWCNT concentration. Moreover, faster recovery was observed for actuation tests carried out in a water bath as compared to tests carried out with a hot plate at the same temperature (∼15 times faster). The shape recovery of TPU and TPU/MWCNT composites in a hot water bath was also recorded to be faster (∼5 times) than in a hot air oven, as shown in Figure 6 [80]. Faster recovery times were reported for 3D-printed TPU/MWCNT composites, namely 130 s for a 0.5 wt.% MWCNT load and 90 s for a 1 wt.% MWCNT load, in contrast with a 245 s recovery time of neat TPU, at 80 °C [123]. Similar studies performed with test specimens prepared by injection molding [134] registered slower recovery for all compositions, which underlines the importance of the processing method.

Blends of TPU with PLA or PCL are often reported, aiming to increase the $R_{r}$ and enhance physical crosslinking within the matrix. Huang et al. [75] studied t-SMP of PLA/TPU blends with loads of 5, 6, and 8 wt.% of MWCNT, observing that the recovery and fixity ratio increased for the composite with better dispersion of the MWCNT (6 wt.%) and dropped for higher MWCNT loading, which presented the worst dispersion. Meng et al. [129] studied the influence of the addition of acid-treated MWCNT (COOH-MWCNT) in a TPU/PCL blend. The COOH-MWCNT were incorporated in TPU by in situ polymerization and further mixed with PCL through melt extrusion and melt spinning; the resulting fibers presented a homogeneous distribution of COOH-MWCNT for contents below 5 wt.%, above which nanotube agglomeration was high, hindering the mechanical properties. A homogeneous distribution of COOH-MWCNT contributed to a higher $R_{r}$ of the composites compared to the TPU/PCL blend, increasing from 83% (TPU/PCL fiber) to 91% (for the 1 wt.% COOH-MWCNT composite). Processing through multilayer co-extrusion resulted in a better shape-memory response to direct heating of TPU/PCL composites with MWCNT, compared to melt-extruded TPU/PCL-MWCNT composites. Multilayered composites present high phase continuity and abundantly continuous interfaces; both $R_{f}$ and $R_{r}$ could be further increased through layer multiplication [63].

The shape-memory characteristics of TPU/ABS blends with pristine and fMWCNT (OH-MWCNT and COOH-MWCNT) were analyzed by Memarian et al. [128]; the authors found that the shape recovery of the nanocomposite containing pristine MWCNT was more effective than the nanocomposites with fMWCNT; however, regarding $R_{f}$, the nanocomposites filled with fMWCNT showed better performance. Ehteramian et al. [131] prepared TPU and PVC blends by solvent casting, adding MWCNT, COOH-MWCNT, and PCL-functionalized MWCNT (PCL-MWCNT) at a 0.5 and 1 wt.% filler load. The TPU/PVC (60:40) composites showed higher $R_{r}$ and $R_{f}$ for fMWCNT in comparison to the pristine one, in the order PCL-MWCNT > COOH-MWCNT > MWCNT, with an increase up to 15% in the $R_{f}$ and 4% in the $R_{r}$, attributing this result to a better dispersion of the fMWCNT.

The use of hydrogels as SMP is frequently reported, such as polyaniline (PANI) and polyvinyl alcohol (PVA) [135,136], or derived from natural sources such as chitosan [137], the former being an electrically conductive and, also, a thermoresponsive polymer. PVA/CNT composites were reported as SMP [115,116,138,139]. Du et al. [117] reported an increase in the recovery time of PVA/COOH-MWCNT due to restrictions of the soft segment’s mobility. Nevertheless, a large increase in $R_{r}$ was observed, of 63% (neat PVA) up to ∼100% (PVA with 4 wt.% COOH-MWCNT) due to CNT interaction with the PVA hard segments. Heidarshenas et al. [116] studied PVA aerogel composites (with COOH-MWCNT at contents up to 3 wt.%), observing an increase in response time with the increase of COOH-MWCNT content, which contributes to a lower chain mobility of PVA. Poly(N-isopropylacrylamide) (PNIPAAm) is a thermoresponsive polymer with applications as soft actuators [140], and its composites with CNT were studied for applications as actuators [141,142,143,144].

### 3.2. Indirect Heating

As mentioned in Section 2, CNT and Gr-derived materials are characterized by high electron mobility due to the conjugated π orbital system, which extends along the hexagonal network of $sp^{2}$ hybridized carbon atoms. CNT act as electron carriers, transforming energy from phonons or electrons into heat. Lower defects on the walls make SWCNT more conductive than MWCNT, displaying different levels according to their chirality [35,145,146]. Thus, these Gr-derived fillers with a high aspect ratio and low density are interesting for the development of polymer matrix composites, aiming at electrothermal and/or photothermal response [12,13,15,20,21,59,67,147,148,149,150,151,152,153,154,155,156,157].

#### 3.2.1. Electrically Driven

The properties and morphology of CNT enable the assembly of an electrically conductive path inside an insulating polymer at low CNT loading, provided adequate control of the CNT dispersion and polymer choice is assured [36,158]. The percolation threshold ($\phi_{c}$) of composites can be estimated according to a percolation power scaling law expressed in Equation (4) [159]:

(4) $$\sigma =\sigma_{0}{\left(\phi -\phi_{c}\right)}^{t}$$

where σ is the electrical conductivity of the composite, $\sigma_{0}$ the electrical conductivity of the filler, ϕ the filler load, and *t* the critical exponent, which reflects the dimensionality of the system [36].

In [133], Koerner et al. [133] proposed an electrothermal mechanism to describe shape deformations that occur in composites with CNT. When a current is passed through a conductive CNT network, it generates heat by Joule heating, which affects the entropy of the polymer, which may further induce the molecular motion of the polymer chains. If a power source is provided, the conversion of electrical energy into thermal energy will occur due to the electrical resistance of the CNT. Equation (5) describes the electrical power loss (*P*, in J·s^−1^), proportional to the tension drop (*V*) and inversely proportional to the resistance (*R*) of a system. The higher the power loss is, the more energy will be converted into heat:

(5) $$P=\frac{V^{2}}{R}$$

**Figure 7:**
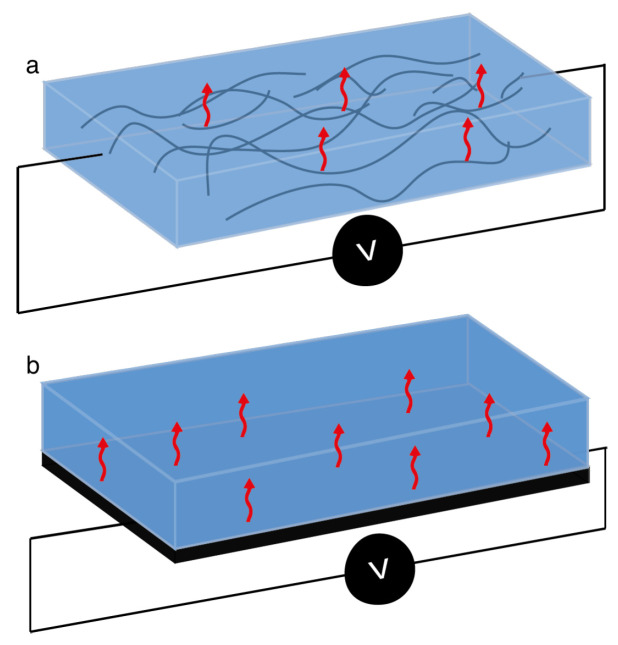
Schematic representation of CNT electrically conductive paths: (**a**) network formed within a polymeric matrix; (**b**) added layer of bulk CNT and SMP matrix.

The power requirements for designed systems will depend on the temperature of the thermal transition necessary to initiate deformation (i.e., $T_{trans}$). It is then possible to fine-tune the time to actuation and the tension applied to a system.Conductive networks may be assembled within a polymer through the different mixing/dispersion techniques or by layering/coating polymers with tightly packed CNT, such as coating with a CNT dispersion (CNT inks) or integrating CNT buckypapers, as schematized in Figure 7a and Figure 7b, respectively.

Fast deformations may be achieved at the expense of increased tension. The number of cycles an electrothermal SMP (et-SMP) can endure is finite, as the internal heat will eventually cause molecular chain degradation. This parameter is not often taken into account when evaluating et-SMP performance. Studies report from 10 cycles, for an et-SMP based on a poly(styrene-butadiene-b-styrene) (SBS)/LDPE/MWCNT composite actuated at 80 V [119], up to 100 cycles, for an et-SMP based on EVA spray-coated with MWCNT actuated at 15 V [160].

Studies concerning et-SMP based on thermoplastics outnumber those with thermoset polymers. Table 5 lists a summary of research studies reported for et-SMP based on thermoset polymers and CNT. Electrical resistivity varies from 8.9 × 10^−2^ [161] to 1 × 10^4^ Ω·cm [90]. The most common system is based on pristine MWCNT and an ER matrix fabricated by impregnation. The composite presenting the lowest resistivity is composed of a hybrid network of CF and CNT (hCNT), in a buckypaper impregnated with ER. Concerning composites with a single filler, the lowest resistivity value of 3.16 × 10^−1^ Ω·cm was reported for a MWCNT buckypaper (40 wt.%) impregnated with ER [162]. The faster recovery time reported is 8 s for an applied tension of 17 V, observed for a composite of CNT foam (1.08 wt.%) with ER, with a resistivity of 7.52 × 10^−1^ Ω·cm, and $T_{trans}$ of 100 °C.

The impregnation of pre-assembled CNT structures seems the most effective method for et-SMP production. However, the scalability of the process is an important factor, as it may be a drawback for industrial applications and environmental concerns due to the use of solvents and a two-step approach (i.e., assembly of CNT structures followed by impregnation with polymer resin). Jung et al. [163] studied the dispersion of 4 wt.% HOOC-MWCNT in TPU through solvent casting, in situ polymerization, and crosslinking polymerization, observing enhanced dispersion and higher shape recovery and fixity rates for composites obtained by cross-linking polymerization under 40 V applied tension.

**Table 5:** Electrothermal SMP with CNT. Overview of processing methods, resistivity of composites, and tension and recovery time of thermoset SMP with CNT.

CNT Type	Content	Polymer	Processing	Resistivity (Ω·cm)	Applied Tension (V)	Recovery Time (s)	*T_trans_* (°C)	Year	Reference
MWCNT	0.3 wt.%	AR	Three-roll mill + 3D printing (DLP)	3.70 × 10^3^	180	20	36	2017	[164]
MWCNT	0.2 wt.%	ER	Three-roll mill + curing	1.00 × 10^4^	126–265	NA	75–176	2022	[90]
	30 wt.%		Impregnation + curing	4.71 × 10^3^	12	22	30–40	2021	[165]
	40 wt.%			3.16 × 10^−1^	4.6	20	54	2020	[162]
CNT	1.08 wt.%			7.52 × 10^−1^	17	8	100	2016	[166]
	1.08 wt.%			7.52 × 10^−1^	8 and 2	60	100		
	0.2 wt.%			7.75 × 10^−1^	10	10	∼106	2016	[167]
hCNT	0.08 g (CNT)			9.30 × 10^−2^	12	45	110	2013	[168]
	0.6 g (CF)								
	0.2 g (CNT)			8.60 × 10^−2^	10	40	110	2013	[161]
	0.6 g (CF)								
hMWCNT	0.4 wt.% (MWCNT)	hydro ER	Stirring + curing	1.00 × 10^3^	225	2100	∼71	2012	[169]
	1.5 wt.% (CB)								
SWCNT	4 wt.%	PU	Solvent casting	4.00	30	90	∼50	2011	[170]

AR—acrylic resin; ER—epoxy resin; PU—Polyurethane; hCNT—hybrid CNT; hMWCNT—hybrid MWCNT.

Table 6 lists et-SMP based on thermoplastic polymer composites with CNT reported in the literature. The majority of the studies use MWCNT as the conductive phase, with PLA as the most studied polymer matrix, which is interesting since PLA is not an elastomer. However, PLA is tested for SMP as a single matrix or blended with other polymers. The preferred processing route for composite preparation is melt mixing, and the resistivity values reported range from 1 × 10^−3^ to 1.87 × 10^7^ Ω·cm.

Raja et al. [73] studied PLA/TPU with pristine and *O*_3_–MWCNT; the addition of modified CNT resulted in the formation of PLA/TPU nanocomposites with higher electrical and thermal conductivity compared to pristine CNT-loaded et-SMP, resulting in a 3× faster recovery.

Liu et al. [171] used CNT/PLA filament to 3D print an et-SMP, studying the influence of the printing speed, layer thickness, and raster angles, concluding that a slower printing speed together with a thicker layer (0.33 mm) and a raster angle of 0° benefit a faster electrothermal response of the et-SMP and lead to lower electrical resistivity (∼60 Ω·cm). Dong et al. [172] studied 3D-printed et-SMP structures with different filling directions, observing that this affects the heat distribution.

**Table 6:** Electrothermal SMP with CNT. Overview of processing methods, resistivity of composites, and tension and recovery time of thermoplastic SMP with CNT.

CNT Type	Content	Polymer	Processing	Resistivity (Ω·cm)	Applied Tension (V)	Recovery Time (s)	*T_trans_* (°C)	Year	Reference
MWCNT	-	EVA	Spray coating	1.05	15	-	70	2021	[160]
	10 wt.%		Ball milling + compression molding	6.01 × 10^−1^	-	-	-	2022	[173]
	10 wt.%		Melt mixing	21.3	30	-	70		
fMWCNT	2 wt.%		Ultrasound adsorption	46.0	50	30 to deformation	77	2023	[174]
	3.77 wt.%			1.64 × 10^−2^	60	19 to deformation	74	2022	[175]
CNT	5 wt.%	EVA/PCL (60:40)	Melt mixing + compression molding	20.5	20	24	94	2016	[69]
					30	12	94		
CNT	10 wt.%	PAEK	Solvent casting	1.70	25	27	131	2020	[103]
	15 wt.%			9.84 × 10^−1^	20	60			
MWCNT	8 wt.%			18.0		∼115	-	2022	[176]
hMWCNT	MWCNT (2 wt.%) and CB (6 wt.%)			11.0	60	∼100			
	MWCNT (4 wt.%) and CB (4 wt.%)			16.0		∼130			
	MWCNT (6 wt.%) and CB (2 wt.%)			19.0		∼115			
MWCNT	1 wt.%	PBS/PCL	Solvent casting	1.60 × 10^3^	75	45	67	2017	[177]
MWCNT	0.5 wt.%	PBS/PEG	In situ polymerization	2.26 × 10^2^	80	313	38.3	2013	[178]
	1 wt.%			14.2	60	57	41.5		
SWCNT	3 wt.%	PCL-Py	Solvent casting	2.11	50	20	65	2019	[111]
MWCNT	5 vol.%	PCL/EOC	Melt mixing + compression molding	16.7	30	30	57	2019	[179]
MWCNT	15 vol.%	PE/PCO (30:70)	Melt mixing	1.00	150	120	-	2014	[180]
MWCNT	40 wt.%	PEG	Solvent casting	1.75	27	15	∼50	2022	[181]
fMWCNT	3 wt.%	PLA	Solvent casting	1.87 × 10^7^	40	75	∼54	2018	[49]
fCNT	5 wt.%		Melt mixing + compression molding	1.00× 10^4^	40	25	56	2014	[46]
MWCNT	5 phr			1.00 × 10^4^	60	12	-	2018	[48]
	8 wt.%		Melt mixing	22.0	20	90	66	2022	[172]
				18.5	10	30			
			3D printing	-	35–50	70–90	66		
hCNT	1 wt.%	PLA/ESO	Solvent casting	1.10 × 10^4^	40	75	-	2015	[47]
	2 wt.%			1.90 × 10^2^		35			
	3 wt.%			3.50 × 10^3^		63			
hCNT	CNT (4 wt.%) and rGO (2 wt.%)	PLA/PCL	Solvent casting	1.67 × 10^−3^	80	60	74	2019	[72]
MWCNT	3 wt.%	PLA/PCL (50:50)		1.00 × 10^2^	100	5	∼60	2012	[71]
MWCNT	14 wt.%	PLA/PEU (70:30)	Melt mixing + 3D printing	3.40	13	150	47	2023	[182]
					17	25	61		
MWCNT	10 wt.%	PLA/TPU (10:90)	Melt mixing + compression molding	1.00 × 10^2^	40	40	∼55	2013	[73]
fMWCNT						15			
SWCNT	2 wt.%	PLA/TPU (60:40)		1.00 × 10^4^	-	80	40.5	2019	[50]
hMWCNT	MWCNT (1 phr) and CB (3 phr)	PLA/TPU (70:30)		45.5	30	100	∼70	2018	[76]
	MWCNT (1 phr) and CB (5 phr)			21.0		70			
MWCNT	4 wt.%	PLA/TPU (40:60)		8.00	20	80	∼70	2017	[74]
		PLA/TPU (50:50)		5.00		50			
MWCNT	3 phr	PLA/PPC (30:70)	Melt mixing + compression mounding	1.00 × 10^−1^	20	90	∼50	2016	[183]
					30	30			
MWCNT	2 vol.%	POE	Ball milling + compression molding	21.7	36	18	∼60	2019	[184]
MWCNT	0.25 wt.%	PVA	Stirring + freezing–thawing	2.33 × 10^2^	100	-	53	2021	[115]
MWCNT	0.25 wt.%	PVA/ chitosan (75:25)	Stirring + freezing–thawing	1.01 × 10^2^	100	-	53	2021	[115]
MWCNT	3 wt.%	SBS/LDPE	Melt mixing + compression molding	1.00 × 10^4^	60	118	∼110	2017	[119]
	4.5 wt.%			10.01	40	162			
	6 wt.%			1.13	20	145			
	7.5 wt.%			1.00		130			
MWCNT	10 phr	SIS/PEO (50:50)	Melt mixing + compression mounding	12.0	5.5	300	75	2023	[120]
	5 phr			3.77 × 10^2^	25	210			
SWCNT	2.5 wt.%	TPI	Solvent casting + compression molding	11.9	30	250	∼40	2023	[121]
CNT	0.5 to 5 wt.%	TPU	Melt mixing + compression molding	1.00 × 10^−3^	50	60	-	2015	[127]
MWCNT	2.5 wt.%	TPU/PCL	Melt mixing + multilayer coextrusion	10.0	30	60	56	2020	[63]
				1.00 × 10^2^	60	20			
CNT	4 wt.%		Solvent casting	5.00 × 10^−2^	100	120	62	2019	[185]
fMWCNT	10 wt.%	TPU/PVDF (90:10)	Melt mixing + compression molding	5.00 × 10^3^	40	30	-	2014	[85]
				4.00 × 10^2^		15			

EVA—poly(ethylene-co-vinyl acetate); LDPE—low-density polyethylene; mPEg-AA—Acrylic acid-functionalized metallocene polyethylene; PAEK—polyaryletherketone; PE—Polyethylene; PLA—poly(lactic) acid; TPU—Thermoplastic polyurethane; PVA—polyvinyl alcohol; SBS—poly(styrene-*β*-butadiene-*β*-styrene); SIS—poly (styrene-*β*-isoprene-*β*-styrene); PEO—Polyolefin Elastomer; TPI—Trans-1,4-polyisoprene; ABS—Acrylonitrile buradiene styrene; PCL—poly(ϵ-caprolactone); PEG—Polyethylene glycol; PVC—Polyvinyl Chloride; UHMWPE—Ultra-High-Molecular-Weight Polyethylene; hMWCNT—hybrid MWCNT; fCNT—functionalized MWCNT; fMWCNT—functionalized MWCNT; hSWCNT—hybrid SWCNT.

#### 3.2.2. Light-Driven

Light-driven actuation powered by CNT may follow a photothermal effect where a broad range of lasers/lamps can trigger CNT-loaded SMP deformations due to their broad absorption in the EM spectrum. Radiation absorption will heat the CNT, which, in turn, will transfer heat to the polymer matrix, resulting in stored strain release, hence being capable of a fast shape deformation [12,15,152,156,186].

The radiation heat transfer can be determined by the Stefan–Boltzmann law, as described in Equation (6):

(6) $$Q=\sigma \epsilon AT^{4}$$

where *Q* is the radiated energy by the body, σ is the Stefan–Boltzmann constant (5.67·10^−8^ W·m^−2^·K^−4^), ϵ is the emissivity of the body (values ranging from 0 to 1, where 1 is assigned to a black body), *A* is the surface area of the body, and *T* is the absolute temperature.

Several studies reporting thermoset photothermal SMP (pt-SMP) use ER as the matrix and MWCNT as fillers, processed through stirring plus curing methods. Recovery times are frequently below 35 s, lower than those reported for thermoset t-SMP and et-SMP (90 and 78 s, respectively). A minimum recovery time of 13 s was obtained for a ER/benzoxazine resin (BR) matrix with 0.5% of MWCNT, under near infra-red (NIR) light [187]. Activation temperatures range between 50 °C [188] and 110 °C [189]. Although systems actuated at NIR are the most common, studies have shown actuation under radio frequency (RF), at 13.56 Hz with ER loaded with MWCNT [189,190]. Microwave (MW) radiation is also used to trigger pt-SMP based on ER with MWCNT, with the pt-SMP being tested in the power range of 60 to 150 W, typically for one-way SMP [191,192]. Table 7 provides an overview of the works reported for thermoset matrices and CNT.

Thermoplastic polymers have been used to produce composites with pristine or fMWCNT for pt-SMP preparation. EVA and TPU are frequently selected as the matrix, with 0.1 wt.% MWCNT loads showing effectiveness in response to NIR [177] and Visible (Vis) [6] wavelengths, recovering the initial shape within 15 s of exposure. pt-SMP have the advantage of remote actuation and also of reversible deformations (Figure 2). With deformation to the temporary shape occurring mostly below 25 s, the fastest deformation time is 4 s for PLA with 0.5 wt.% MWCNT under NIR [195]. Thermoplastic pt-SMP described in the literature actuate under NIR and Vis, with $T_{trans}$ ranging from 30 °C [7] to °C [196]. Table 8 summarizes the thermoplastic pt-SMP reported in the literature, accounting for the EM region used to induce actuation, the power of the laser applied to the sample, the time for deformation and/or recovery, and the transition temperature of the composites selected for the deformation to occur.

Koerner et al. [133] produced pt-SMP of TPU composites with MWCNT, obtaining a composite that contracts and exerts ∼19 J, able to lift 60 g for a height greater than 3 cm when stimulated in the NIR region. Czanikova et al. [6] used both nanoindentation and Atomic Force Microscopy (AFM) to characterize shape deformations in a Braille element, by applying different currents (150, 200, and 300 mA) using red diodes (627 nm) and blue diodes (470 nm); they observed changes in height of EVA composites with 0.1 wt.% of MWCNT, when switching the diodes on and off. A faster response was observed for higher power, i.e., when a higher current was applied. Later, Czanikova et al. [200] studied the photothermal actuation of EVA nanocomposites containing 0.1 wt.% and 3 wt.% of MWCNT and 3 wt.% of SWCNT. Nanocomposites with 0.1 wt.% MWCNT exhibited stresses between 33 and 165 kPa as a function of the red light source (670 nm) without damage, reversible actuation, and high optical-to-mechanical energy conversion factors of 26 and 55 MPa·W^−1^, illuminated for 10 and 30 s. Lai et al. [197] investigated the remotely triggered shape-memory properties of natural rubber (NR) and carnauba wax (CW) bio-based blends and their nanocomposites with MWCNT. They assigned $T_{trans}$ as the $T_{m}$ of the wax and used an NIR laser for actuation, observing shape recovery within 120 s and that the presence of MWCNT prevented the coalescence of dispersed wax domains in the NR.

**Figure 8:**
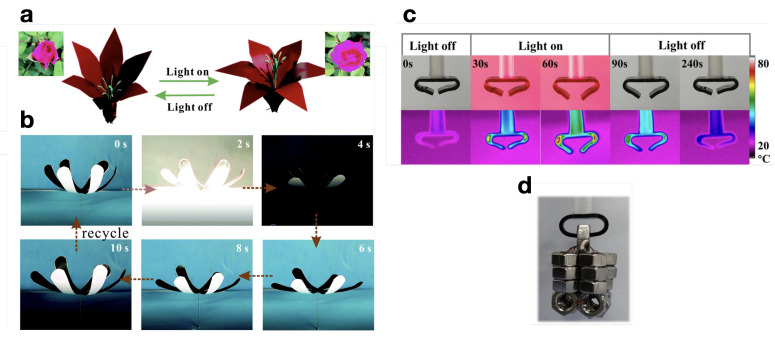
(**a**) Schematic representation and real images of the flower blooming process; (**b**) phototriggered shape change of a 3D-printed flower mimicking a flower blooming: blooming during illumination and shape recovery after turning off the light source. Reprinted with permission from Hua et al. [195]. Copyright 2018, Royal Society of Chemistry. (**c**) Digital and corresponding IR camera images of a gripper based on a PEO/PW composite with 0.1 wt.% MWCNT, triggered by IR light, switched on and off; (**d**) image of the gripper in (**c**) grabbing weights. Adapted with permission from Xu et al. [196]. Copyright 2022, American Chemical Society.

Composites formed by bi-layers of a PLA/MWCNT composite and paper are reported by Hua et al. [195]. The bi-layer system is capable of reversible motion, i.e., the deformation is triggered by switching on a light source (NIR lamp), and it recovers the initial shape when the light is turned off (Figure 8a,b). Conversely, composites formed by PLA/MWCNT show an irreversible response; deformation occurs with incident light, but sustains it when the light is off. Xu et al. [196] designed a gripper based on polyolefin elastomer (PEO)/paraffin wax (PW) composites loaded with 0.1 wt.% MWCNT (Figure 8c,d). The reversible actuator takes 60 s to deform to its temporary position (open) after a light is switched on (IR lamp) and recovers its initial shape in 180 s when the lamp is turned off. Figure 8c depicts IR camera images showing the photothermal response powered by MWCNT, with the gripper reaching up to 80 °C, close to $T_{trans}$ of the nanocomposite.

**Figure 9:**
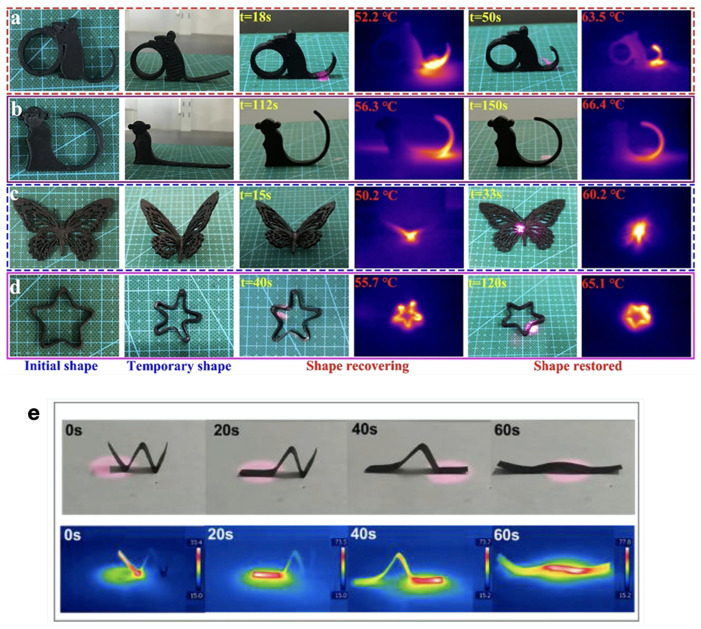
Photos of different 3D-printed models showing one-way shape-memory NIR-induced recovery: (**a**) mouse model, (**b**) small monkey model, (**c**) butterfly model, and (**d**) pentagram model of NIR-induced shape-memory performance. Reprinted with permission from Bi et al. [83]. Copyright 2020, Elsevier. (**e**) Localized shape recovery of a “W-shaped” stripe of a PCL-Py/SWCNT composite, recorded using a digital camera and an IR camera. Adapted with permission from Xiao et al. [111]. Copyright 2019, Elsevier.

A stress-recovery mechanism powered by a xenon lamp (>900 nm) is reported for TPU nanocomposites with fMWCNT and graphene oxide (GO), with a concentration of 0.25 wt.% of fMWCNT, by Feng et al. [86]. The stress-recovery process of the pt-SMP composite enables lifting a 107.6 g weight to a 4.7 cm height in 18 s under IR light stimulation, with an estimated energy density of 0.63 J/g. Bi et al. [83] prepared a blend of TPU/PCL (∼60:40) mixed with dopamine (DA) modified MWCNT (3 wt.%), melt-extruded a filament, and 3D-printed different shapes. The photothermal actuation was studied using NIR light (808 nm), observing interfacial enhancement due to the DA modification of the MWCNT. Moreover, the recovery times measured from the programmed to the permanent shape were between 33 and 150 s, depending on the shape (Figure 9). Xiao et al. [111] prepared “W-shaped” stripes of a pyrene-containing poly(ϵ-caprolactone) (PCL-Py) composite with 3 wt.% of SWCNT. The shape was controlled by localized irradiation with NIR light, showing recovery of the permanent shape in 60 s by shining light on three different areas of the “W-shaped” sample (Figure 9e). Hsu et al. [201] report on a pt-SMP of a polystyrene (PS) composite with acid-treated CNT (0.5 to 4 wt.%), showing that the recovery time of the one-way SMP decreases with increasing concentration of filler.

It was observed that, in general, for pt-SMP actuation, a homogeneous distribution of CNT in the composite is more important than their dispersion within the matrix.

## 4. Other Types of Actuation of SMP with CNT

**Figure 10:**
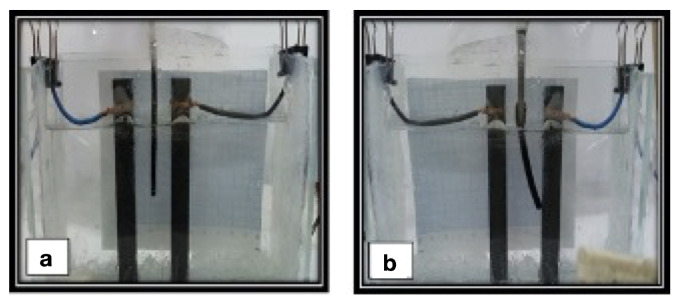
Electrical activity of PVA/CNT hydrogel in NaCl (**a**) at the initial position, and (**b**) after an applied voltage of 40 V. Adapted with permission from Pirahmadi et al. [115]. Copyright 2020, John Wiley and Sons.

In 1999, Baughman et al. [202] described SWCNT actuators using dimensional changes in covalent bond directions caused by charge injection, originating from quantum chemical and electrostatic double-layer effects, showing their potential for the efficient and direct conversion of electrical into mechanical energy. Approximately 20 years ago, Landi et al. [203] produced composite actuators based on Nafion reinforced with purified SWCNT, aiming at converting electrical into mechanical energy through an electrochemical method [204]. An electrical conductivity of 40 Scm·^−1^ was achieved for composites with 18% (*w*/*w*) SWCNT, allowing the measurement of cantilever displacement in a LiCl solution by the application of frequencies up to 50 Hz, concluding that SWCNT composites performed better than metal-doped Nafion films [203]. Tahhan et al. [205] coated SWCNT mats with PANI, an electrically conductive polymer, finding a synergistic effect between the two, the composite presenting higher electrical conductivity than individual CNT mats or the neat polymer, translating into an enhanced electrochemical efficiency. In terms of actuation response, the composites showed enhanced actuation compared to CNT mats, but similar actuation to neat PANI, since the PANI redox processes dominate actuation. However, the composite allowed higher strain to be maintained at higher applied stresses. In 2021, Pirahmadi et al. [115] reported a PVA/CNT hydrogel with electrochemical actuation in a NaCl solution, as depicted in Figure 10. A three-component system tested by the same authors with extra chitosan showed higher electrical conductivity and enhanced electrochemical actuation.

**Figure 11:**
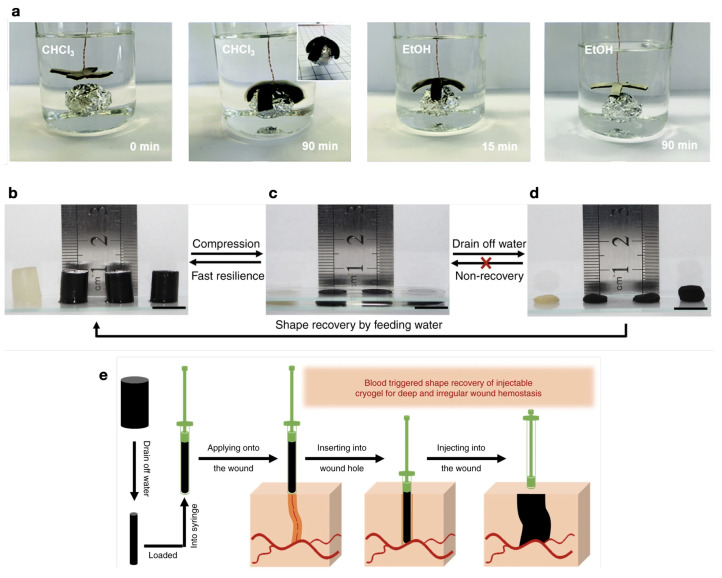
(**a**) Deployed and folded configurations of the PCL-PCL/MWCNT bi-layer film before and after immersion in CHCl_3_. shape-memory properties of MWCNT-filled cryogels. Adapted with permission from Toncheva et al. [210]. Copyright 2017, Royal Society of Chemistry. (**b**–**d**) shape-memory property of the MWCNT-filled cryogels: fast resilience and macroscopical shape-memory property (scale bar: 1 cm); (**e**) schematics of the application of hemostatic injectable shape-memory cryogel in a deep and irregularly shaped wound model. Adapted from Zhao et al. [211].

Electromechanical actuation can be achieved in polymer/CNT composites, recurring to both polymer (such as polyvinylidene fluoride (PVDF)) and CNT piezoelectric effects [206,207]. This actuation mechanism is based on the direct transformation of an electrical stimulus into a mechanical deformation [208].Water-triggered actuation was observed for TPU/MWCNT composites by Luo et al. [209], attributing the actuation to the plasticizing effect of water molecules that depress the $T_{g}$ of TPU. Moreover, the presence of CNT facilitated water diffusion, enhancing shape recovery. Toncheva et al. [210] described a bi-layer solvent-responsive SMP, with PCL and PCL/MWCNT layers; an increased rigidity and a decreased degree of swelling of the PCL/MWNCT layer contributed to controlled bending movement of the bi-layer actuator, as depicted in Figure 11a), which shows the actuator grabbing an object, in $CHCl_{3}$, and releasing it when transferred to an EtOH solution, getting back to its initial position with time. Zhao et al. [211] studied cryogels with MWCNT for applications in wound healing, with a shape-memory ability, recovering the initial state when in contact with water and blood, as illustrated in Figure 11b–e. The composites presented a 100% $R_{r}$ and a response time of 1 s, both with and without CNT, but the presence of CNT endows composites with better homeostatic capacity, better in vivo wound healing, and the potential for drug release through a photothermal effect. Recently, Wang et al. [212] reported PVA/CNT fibers capable of water-driven actuation, rotating and contracting in water. However, the contribution of MWCNT to the mechanism needs to be clarified.

## 5. Multifunctionality in SMP-CNT Composites

### 5.1. Self-Healing

Prolonging lifespan, enhancing reliability, and decreasing waste are desirable features for materials. Vastly researched over the past years, the self-healing properties of polymers often come in hand with research on the SME. Of the different mechanisms that can trigger self-healing, those based on the thermal expansion of polymer chains are the same that trigger t-SMP, et-SMP, and pt-SMP, i.e., self-healing can be achieved through direct or indirect heating, through an SME. Moreover, the generated heat on the CNT can also contribute to the thermal activation of reversible bonds [213,214,215]. Table 9 presents a few examples of SMP with CNT that display a self-healing ability, alongside with the self-healing mechanism.

**Figure 12:**
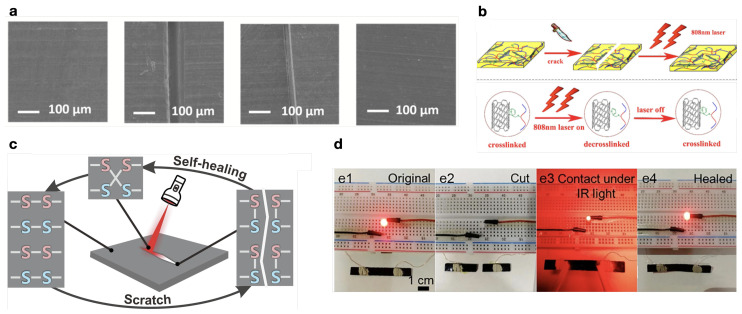
(**a**) Electrical self-healing of a modified PCL with MWCNT by the formation of new covalent bonds. Adapted with permission from Houbben et al. [216]. Copyright 2023, Elsevier. (**b**) Scheme of the healing process, and the reaction occurring during healing of an NIR-healed SBS with MWCNT. Reprinted with permission from Bai and Shi [219]. Copyright 2017, American Chemical Society. (**c**) Illustration of the self-healing mechanism by disulfide exchange. Adapted with permission from Miao et al. [193]. Copyright 2022, Elsevier. (**d**) Digital photos of the circuit constructed by a PPC/MWCNT sheet and an LED lamp at different states; (**e1**) the lamp is lit at the initial state; (**e2**) the composite sheet is cut into two parts, and the lamp is extinguished; (**e3**) the two parts of the sheet are connected under IR light; (**e4**) healed sheet by IR irradiation, which rebuilds the circuit to light the LED. Adapted with permission from Cui et al. [220]. Copyright 2020, Elsevier.

Electrically triggered SMP with self-healing ability are reported in the literature. Fan et al. [218] prepared a TPU/SBS blend with MWCNT, achieving self-healing via reversible C-ON covalent bonds. Houbben et al. [216] obtained self-healing for PCL capped with maleimide and furan groups, allowing thermal triggering of a retro Diels–Alder (rDA) reaction, forming new covalent bonds (Figure 12a). Orozco et al. [217] studied the activity of furan-grafted polyketone (PK) with MWCNT also via an rDA reaction. Ren et al. [72] report the self-healing response of a PLA/PCL blend with CNT and rGO.SMP with light-triggered self-healing ability were achieved by Xiao et al. [221], for PVA hydrogels with MWCNT and nanocellulose. Xie et al. [106] investigated the response of EVA composites with MWCNT and MXenes, both fillers presenting great light-to-thermal conversion. Bai and Shi [219] investigated the response of a furan-modified SBS with MWCNT, where the heat generated by the MWCNT triggers an rDA reaction, enabling chemical rearrangement and, therefore, healing (Figure 12b). Dai et al. [222] produced a PVA composite with pyrene-modified CNT. Acrylic acid-functionalized metallocene polyethylene (mPE-g-AA) with MWCNT was prepared by Lai et al. [109], while Xu et al. [199] reported the activity of a TUP/PCL blend with MWCNT. Miao et al. [193] utilized an ER with MWCNT, where the nanotubes assist the photothermal conversion for the dynamic disulfide bonds, which undergo reversible exchange and release stress, resulting in self-healing (Figure 12c). Cui et al. [220] attained self-healing for biodegradable poly(propylene) carbonate (PPC) composites with MWCNT (Figure 12d). Using light sources to trigger both the SME and self-healing has the advantage of remote and selective stimulation compared to other thermal mechanisms.

### 5.2. Sensing

CNT composites may be prepared in a range of compositions within the electrical percolation threshold, below or above it. Thus, CNT composites are good candidates for strain sensing, where a change in resistance may induce a deformation. The multifunctionality of SMP with strain-sensing ability has been reported. Flexible sensors based on electrically conductive polymer composites enable high strain sensing, helping to overcome the limited detection range of traditional strain sensors (∼5%) [223].

**Figure 13:**
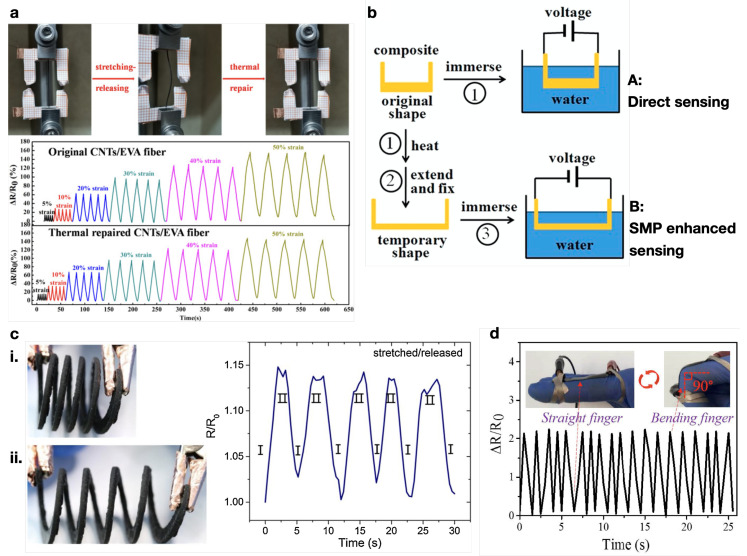
(**a**) Photos of EVA/CNT fibers at different states: the initial state, after 1400 stretch–release cycles, and after thermal treatment; relative resistance changes of the original and thermally repaired EVA/CNT fibers with different strains at 30 mm·min^−1^. Adapted with permission from Li et al. [107]. Copyright 2020, American Chemical Society. (**b**) Schematic illustration of water-sensing processes for direct and SMP-enhanced sensing. Adapted with permission from Luo et al. [225]. Copyright 2015, Elsevier. (**c**) Images of a stretchable spring structure i. at rest and ii. in stretched positions, and the changes in resistance of a gauge during five cycles. Reprinted with permission from Mu et al. [164]. Copyright 2017, Elsevier. (**d**) Relative resistance variations vs. time for a TPU/MWCNT SMP finger-like strain sensor bending and stretching. Adapted from Wu et al. [224].

EVA SMP composites, with COOH-MWCNT [107,174] and MWCNT with MXene [106], were studied as strain actuators. Both Li et al. [107] (Figure 13a) and Xie et al. [106] report on SMP with the sensing and self-healing ability of EVA composites, with Xie et al. [106] attaining up to 5000 cycles of sensing after repair with NIR. Xiao et al. [221] tested composite PVA hydrogels for wearable strain sensors under radiation.

PU-based SMP composites with MWCNT and nanocellulose [224] (Figure 13d) and MWCNT [225] were studied for their strain-sensing and shape-memory-enhanced water-sensing ability (Figure 13b), respectively. Xu et al. [196] propose a self-sensing actuator for strain and temperature made by PEO/PW coated with MWCNT, where the MWCNT form a conductive path for sensing, whereas the PEO/PW acts as the shape-memory material. Both water and temperature SMP sensors were produced by Lu et al. [226], with ER composites with fMWCNT.

Mu et al. [164] prepared a DLP 3D-printed acrylic resin (AR)-/MWCNT-based capacitor and strain sensor (Figure 13c). Kernin et al. [227] developed kirigami-inspired SMP of PS with CNT veils that are capable of self-sensing at different folding angles, induced by a change in electrical resistance.

## 6. Conclusions

The design of SMP/CNT may differ based on the specific conditions under which they are intended to operate. For SMP with higher temperature requirements, thermoset matrices may be preferred due to their higher $T_{g}$; however, the high crosslinking degree of the polymer may increase stiffness and limit complex movements. On the other hand, thermoplastic polymers enable actuation at low temperatures, depending on the polymer choice.

The addition of CNT to t-SMP composites mainly results in a higher fixity of the temporary shape due to the increase in mechanical strength; however, it may also induce a lower deformation recovery. To achieve optimal results, the filler loading, dispersion, and distribution of CNT in composites must be considered.

Typically higher than required for sensing devices, et-SMP should display high electrical conductivity to reach $T_{trans}$ at low power; this requires a well-dispersed CNT network within the polymer, optimized for a load that does not negatively affect the mechanical properties. Careful selection of composite processing methods is, therefore, necessary. While solvent casting and in situ polymerization deliver good control of the composite properties, their scalability has limitations due to the use of undesirable solvents. Thermoplastic composite processing methods based on melt mixing are ideal for large-scale production and may yield adequate CNT dispersion under controlled conditions. et-SMP are in high demand in industries such as robotics for developing programmable soft actuators.

Remotely and selectively triggered SMP can rely on a photothermal mechanism (pt-SMP). Unlike et-SMP, where the distribution is the key to achieving a homogenous and reproducible response, preparing pt-SMP composites does not require excellent dispersion control. Low loading of CNT can kick-start localized heating of the polymer chains, resulting in a fast and often reversible actuation process. pt-SMP can mimic processes present in nature and are valuable for medical and tissue engineering applications.

A few researchers have addressed other mechanisms of actuation of CNT-filled SMP. The piezoelectric response of CNT and Gr is of interest for micro- and nano-electromechanical systems and could benefit from the flexibility provided by polymer matrices. CNT actuation can be explored to assemble multi-material systems that rely both on hydrophilic and hydrophobic interactions, such as in multilayer systems.

Self-healing is often achieved with the exact mechanisms that trigger a deformation in SMP, highlighting the potential of SMP as reliable and durable materials. The addition of CNT provides self-repairing through Joule heating to trigger the relaxation of the polymer soft chains, melt and join the materials, or, by providing sufficient thermal energy, allowing covalent bonds to be re-established. Life cycle assessment is essential and should be performed in pursuit of sustainable options. Furthermore, addressing critical challenges in improving the processing routes by moving towards solvent-free approaches and developing novel methods to facilitate the processing of natural polymers and the dispersion of particulate fillers is crucial for the sustainability of these materials. In addition, the recycling of SMP/CNT presents a challenge for developing more eco-friendly options that benefit from the unique properties of CNT.

For the upcoming years, research in SMP/CNT will likely center on the multifunctionality of these materials, including integrated sensing, healing, and shape-memory properties, which can effectively operate in challenging environments. Additionally, these materials can be tailor-made to achieve specific end goals through numerical simulations and the availability of optimized processing routes. Furthermore, actuation mechanisms based on electromechanical and solvent absorption show great promise for micro-/nanoscale applications.

## Figures and Tables

**Table 1:** Different stimuli and actuation mechanisms of SMP, their advantages and disadvantages.

Stimuli	Mechanism of Actuation	Advantages	Disadvantages
Heat [4,5,8,9]	Thermal	Excellent shape-memory effect	Control of local environment
Electric [5,8]	Electro-thermal	Allows remote actuation	Requires high volume filleraddition to reach electrical percolation threshold
	Electro-chemical	Fast actuation	Deformation may alter resistivityvan der Waals forces compromise homogeneity of the matrix
Chemical [5,9]	Adsorption	Targeted design for biological environments	Slow recovery time
	pH changes		
	Chemical bonding		
Magnetic [5,10]	Magneto-thermal	Allows remote actuation No need for a network of adjacent particles	Requires filler addition
	Magneto-mechanical	Possibility of thermoregulatory limit (Curie temperature)	Power requirements
Light [4,5,8]	Photo-thermal	Remote wireless actuation	Requires filler addition or matrix modification
	Photo-chemical	Complex shape shift Fast actuation	

**Table 2:** Electrical and thermal conductivity of CNT and polymers.

	CNT	Polymers
Electrical conductivity	10^3^–10^5^ [31]	10^−15^–10^−8^ [31]
(S·m^−1^)		
Thermal conductivity	10^3^ * [27]	0.1 * [27]
(W·m^−1^·K^−1^)		
Coeff. of thermal expansion	−1.5 [32]	20–200
(ppm·K^−1^)		
Young’s modulus	$1\times 10^{3}$ [33]	1.2 × 10^−2^–4
(GPa)		

* Orders of magnitude.

**Table 3:** Thermoset t-SMP with CNT. Overview of processing methods, shape fixity $R_{f}$, and recovery $R_{r}$ ratios of composites, transition and actuation temperatures, and recovery times.

CNT Type	Content	Polymer	Processing	$R_{f}$	$R_{r}$	$T_{\mathrm{trans}}$	Temperature	Recovery Rate	Year	Reference
	**(wt.%)**			**(%)**	**(%)**	**(°C)**	**(°C)**	**(s)**		
MWCNT	0.1	AR	Three-roll mill + 3D printing (DLP)	65–94	94–99	15–190	15–190	-	2021	[87]
MWCNT	0.5	EPAc/PEGDMA	Stirring + 3D printing	81–97	81–100	67	-	100	2021	[88]
fMWCNT	0.4	ER	Stirring + lamination	96	96	71	91	5	2022	[89]
	0.6			96	96	69	89	5		
	0.4			96	96	73	93	5		
	0.6			96	96	69	89	5		
MWCNT	0.2		Three-roll mill	75–90	>85	68–155	T*_g_* + 10	-	2022	[90]
	0.1		Stirring + curing	-	99	68	68	30	2021	[91]
	0.2			-	95	73	73	70		
	0.5			-	86	77	77	130		
	1			-	93	78	78	80		
	0.1			-	95	70	70	90		
	0.2			-	86	73	73	100		
	0.5			-	83	76	76	150		
	1			-	83	86	86	90		
fMWCNT	0.1			-	∼100	97	107	15	2021	[92]
	0.2			-	∼100	101	111	15		
	0.5			-	∼100	107	117	15		
	1			-	∼100	109	119	20		
hMWCNT	1			80–95	100	43 and 44	80	30	2018	[93]
MWCNT	0.49			98	-	44	53	-	2016	[94]
	0.99			98	-	44	51	-		
	1.47			99	-	45	51	-		
	1.96			99	-	45	51	-		
	0.42 and 0.83		High shear + curing	∼95	∼100	100	100	-	2013	[95]
fMWCNT	0.2	HBPU	In situ polymerization	99.5	99	∼40	60	115	2014	[96]
	1			99.5	99	∼40	60	91		
	2			99.5	99	∼40	60	73		
fMWCNT	0.2	HBPU/ER	In situ polymerization	99	99.3	∼40	60	18	2014	[97]
	1			99	99.3	∼40	60	14		
	2			99	99.3	∼40	60	10		
fMWCNT	0.5	PU	Solvent casting	84	98	78	90	-	2018	[98]
	1			85	95	85	90	30		
	2			88	99	86	90	-		
MWCNT	2		In situ polymerization	87–75	87–78	51	40	60	2013	[99]
fMWCNT	1	PU/PS	In situ polymerization	-	100	85–90	85–100	17	2022	[100]

AR—acrylic resin; EPAc—Epoxy Acrylate; PEGDMA—Polyethylene glycol dimethacrylate; ER—epoxy resin; HBPU—Hyperbranched Polyurethane; PU—Polyurethane; PS—polystyrene; fMWCNT—functionalized MWCNT; hMWCNT—hybrid MWCNT.

**Table 4:** Thermally activated SMP with CNT. Overview of processing methods, shape fixity ($R_{f}$), and recovery ($R_{r}$) ratios of composites, transition and actuation temperatures, and recovery time of thermoplastic SMP with CNT.

CNT Type	Content	Polymer	Processing	$R_{f}$	$R_{r}$	*T_trans_*	Temperature	Recovery Rate	Year	Reference
				**(%)**	**(%)**	**(°C)**	**(°C)**	**(s)**		
hMWCNT	-	EVA	Ultrasonication + soaking	99	94	60	80	-	2022	[106]
fCNT	-			99	96	66	80	-	2020	[107]
MWCNT	1 wt.%	LDPE/EVA (80:20)	Melt mixing + compression molding	69	100	80	140	-	2020	[108]
	3 wt.%			82	100	80	140	-		
MWCNT	0.5 phr	mPE-g-AA + arginine	Solvent casting + compression molding	∼90	82	54	70	-	2022	[109]
MWCNT	5 wt.%	PAEK	Solvent casting	>98	94	171	181	-	2022	[105]
	10 wt.%			>98	95–99	171	181	12		
	15 wt.%			>98	78	171	181	-		
CNT	10 wt.%			-	∼100	131	141	10		
hMWCNT	-	PCL	Solvent casting	99	96	41	41	22	2014	[110]
SWCNT	1, 2, and 3 wt.%			∼99	87–96	43–55	60	-	2019	[111]
fMWCNT	5 wt.%			-	∼95	-	55	20	2010	[112]
hSWCNT	-		Electrospinning	84	92	∼45	46	120	2012	[113]
MWCNT	14 wt.%	PE	Melt mixing + compression molding	-	∼100	37	60	4800	2018	[114]
SWCNT	2 wt.%	PLA/TPU (60:40)	Melt mixing + compression molding	∼100	30	41	80	-	2019	[50]
	4 wt.%			∼100	70	43	80	-		
MWCNT	6 wt.%	PLA/TPU (70:30)	High-speed mixer + melt mixing + 3D printing	95–97	86–98	∼65	65	17	2022	[75]
MWCNT	0.25 wt.%	PVA	Stirring + freeze–thaw	-	100	50	53	62	2021	[115]
fMWCNT	1 wt.%		Stirring + electrospinning + freeze–thaw	-	100	-	95	15	2019	[116]
	1.5 wt.%			-	100	-	95	16		
	2 wt.%			-	100	-	95	19		
	3 wt.%			-	100	-	95	22		
	1 wt.%		Solvent casting	-	∼75	-	90	16	2013	[117]
	2 wt.%			-	∼85	-	90	33		
	3 wt.%			-	>90	-	90	36		
	4 wt.%			-	∼100	-	90	40		
SWCNT	>25 wt.%		Wet spinning PVA-SWCNT + MWCNT coating	∼100	-	75	120	-	2015	[118]
MWCNT	0.25 wt.%	PVA/chitosan (75:25)	Stirring + freeze–thaw	-	54	50	53	82	2021	[115]
MWCNT	1.5 wt.%	SBS/LDPE	Melt mixing + compression molding	-	-	∼110	110	240	2017	[119]
MWCNT	1 phr	SIS/PEO (50:50)	Melt mixing	97	86	75	75	240	2023	[120]
	3 phr			98	82	99	75	240		
	5 phr			97	82	98	75	300		
	10 phr			97	74	99	75	600		
SWCNT	0.5 wt.%	TPI	Solvent casting + compression molding	-	100	∼50	100	25	2023	[121]
	1 wt.%			-	100	∼50	100	35		
	1.5 wt.%			-	100	∼50	100	35		
	2 wt.%			-	100	∼50	100	35		
	2.5 wt.%			-	92	∼50	100	40		
CNT	1 phr	TPI/LDPE	Two-roll mill	98	96	108	130	-	2020	[81]
	2 phr			97	95	107	130	-		
	3 phr			97	92	107	130	-		
MWCNT	0.3 wt.%	TPU	Melt mixing + melt spinning	81	86	∼45	-	-	2023	[122]
	0.5 wt.%			97	83	∼60	-	-		
	0.5 wt.%		Melt mixing + 3D printing	-	-	57	80	130		
	1 wt.%			-	-	57	80	90	2023	[123]
	0.5 wt.%		Melt mixing + injection molding	-	-	57	80	40		
	0.5 wt.%			-	-	57	80	28		
	2 wt.%			∼92	∼80	∼57	-	33 and 160	2022	[80]
	1 wt.%			-	99	-	60	60		
	1 wt.%			-	95	-	60	60	2020	[124]
	1 wt.%			-	90	-	60	60		
CNT	1 wt.%			∼99	∼98	-14	25	-	2013	[125]
fCNT	1 wt.%			∼99	∼99	-11	25	120		
fMWCNT	0.5 wt.%		Melt mixing + compression molding	100	100	43	50	3.3 and 42	2022	[126]
	1 wt.%			99	100	43	50	3 and 38		
	1.5 wt.%			99	100	43	50	2.7 and 36		
	2 wt.%			98	100	43	50	2 and 33		
CNT	0.5 wt.%			-	72	-32	25	60	2015	[127]
	2 wt.%			-	72	-31	25	60		
	5 wt.%			-	71	-30	25	60		
MWCNT	1 wt.%		Melt mixing + two-roll mill	95	92	-32	10	13	2020	[104]
	3 wt.%			98	95	-29	10	12		
	5 wt.%			99	92	-28	10	12		
hMWCNT	0.5 wt.%			94	93	-30	10	12		
	1.5 wt.%			98	96	-29	10	12		
	2.5 wt.%			99	96	-27	10	12		
hSWCNT	∼2 wt.%		Ice templating + solvent casting	96	91	∼40	70	23	2019	[82]
hMWCNT	-		Dip coating	89 - 93	83 - 84	77 - 84	90	10	2016	[102]
MWCNT	2 wt.%	TPU/ABS	Melt mixing + compression molding	99	100	-40	25	-	2019	[70]
				99	99	-39	25	-		
fMWCNT	0.5 wt.%	TPU/ABS (80:20)	Melt mixing + compression molding	99	99	-	25	-	2018	[128]
	1 wt.%			99	98	-	25	-		
	2 wt.%			100	98	-	25	-		
	3 wt.%			100	97	-	25	-		
	5 wt.%			99	96	-	25	-		
	0.5 wt.%			98	98	-	25	-		
	1 wt.%			100	98	-	25	-		
	2 wt.%			100	98	-	25	-		
	3 wt.%			100	97	-	25	-		
	5 wt.%			99	97	-	25	-		
fMWCNT	1 wt.%	TPU/PCL	In situ polymerization + melt mixing	-	92	48	70	-	2007	[129]
	3 wt.%			-	87	45	70	-		
	5 wt.%			-	85	45	70	-		
	7 wt.%			-	55	44	70	-		
MWCNT	2.5 wt.%	TPU/PCL (50:50)	Melt mixing	91	77	-	70	-	2020	[63]
	2.5 wt.%			94	80	-	70	-		
	2.5 wt.%			94	83	-	70	-		
	2.5 wt.%			95	86	-	70	-		
MWCNT	3 wt.%	TPU/PEG	Phase inversion	91	81	-	50	-	2018	[130]
	3 wt.%		Melt mixing	75	61	-	50	-		
fMWCNT	1 wt.%	TPU/PVC (30:70)	Solvent casting	87	98	20	50	-	2021	[131]
	1 wt.%	TPU/PVC (50:50)		92	82	55	85	-		
	0.5 wt.%	TPU/PVC (60:40)		77	93	30	60	-		
	1 wt.%			89	96	30	60	-		
	0.5 wt.%			87	96	31	61	-		
	1 wt.%			90	96	35	65	-		
MWCNT	0.5 wt.%			72	92	29	59	-		
	1 wt.%			82	92	30	60	-		
MWCNT	3 wt.%	UHMWPE	Solvent casting + compression molding	95	94	-	115	120	2022	[132]

EVA—poly(ethylene-co-vinyl acetate); LDPE—low-density polyethylene; mPEg-AA—Acrylic acid-functionalized metallocene polyethylene; PAEK—polyaryletherketone; PE—Polyethylene; PLA—poly(lactic) acid; TPU—Thermoplastic polyurethane; PVA—polyvinyl alcohol; SBS—poly(styrene-β-butadiene-β-styrene); SIS— poly (styrene-β-isoprene-β-styrene); PEO—Polyolefin Elastomer; TPI—Trans-1,4-polyisoprene; ABS—Acrylonitrile buradiene styrene; PCL—poly(ϵ-caprolactone); PEG—Polyethylene glycol; PVC—Polyvinyl Chloride; UHMWPE—Ultra-High-Molecular-Weight Polyethylene; hMWCNT—hybrid MWCNT; fCNT—functionalized MWCNT; fMWCNT—functionalized MWCNT; hSWCNT—hybrid SWCNT.

**Table 7:** Overview of processing methods, EM region, power, and recovery time of photothermal SMP based on thermoset polymers and CNT.

CNT Type	Content	Polymer	Processing	EM Region	Heat Flux Density	*T_trans_* (°C)	Recovery Time (s)	Year	Reference
CNT	0.4	ER	Stirring + curing	RF	-	59	-	2011	[190]
MWCNT	2			NIR	18,000	102	60	2022	[193]
hCNT	4		High shear + curing	NIR	-	107	60	2014	[194]
MWCNT	0.5		Stirring + ultrasonication + curing	RF	-	111	35	2017	[189]
MWCNT	0.1	ER/BR	Stirring + curing	NIR	-	102	36	2019	[187]
	0.3				-	107	16		
	0.5				-	97	13		
MWCNT	0.1	ER/CNSL (70:30)	Ultrasonication + curing	NIR	120	∼50	100	2018	[188]
	0.3				120		95		
	0.5				120		65		

ER—epoxy resin; BR—benzoxazine resin; CNSL—Cashew nut shell liquid; hCNT—hybrid CNT.

**Table 8:** Photothermal SMP with CNT. Overview of processing methods, EM region, power, and deformation and/or recovery times of thermoplastic SMP with CNT.

CNT Type	Content	Polymer	Processing	EM Region	Heat Flux Density	*T_trans_* (°C)	Deformation (s)	Recovery Time (s)	Year	Reference
MWCNT	2 wt.%	EUG	Solvent casting + two-roll mill	NIR	-	∼35	-	30–100	2023	[7]
fMWCNT	2 wt.%	EVA	Ultrasound adsorption	NIR	2000	77	-	50	2013	[174]
MWCNT	0.1 wt.%		Solvent casting + compression molding	Visible	-	-	35	65	2012	[6]
					-	-	6	30		
					-	-	14	32		
					-	-	15	15		
MWCNT	0.5 phr	NR/CW (60:40)	Melt mixing + compression molding	NIR	-	∼75	-	120	2019	[197]
MWCNT	0.1 wt.%	PEO/PW	Melt mixing + compression molding	IR	2000	∼80	60	240	2022	[196]
	1 phr	PEO/PW (40:60)	Solvent casting	NIR	200	46	-	90	2019	[198]
	3 phr						-	60		
	5 phr						-	50		
MWCNT	0.1 wt.%	PBS/PCL	Solvent casting	NIR	3200	67	-	15	2017	[177]
SWCNT	3 wt.%	PCL-Py	Solvent casting	NIR	90	65	-	50	2019	[111]
							-	60		
MWCNT	0.5 wt.%	PLA/paper	Solvent casting + melt mixing + 3D printing	NIR	2750	62	4	25	2018	[195]
MWCNT	0.5 wt.%	POE	Ball milling + compression molding	IR	2500	60	15	-	2019	[184]
hMWCNT	0.25 wt.%	TPU	Solvent casting	NIR	300	38	-	18	2013	[86]
MWCNT	1–5 wt.%	TPU/PCL	Melt mixing + compression molding	NIR	-	∼60	-	33	2018	[199]
fMWCNT	3 wt.%	TPU/PCL (60:40)	Solvent casting + Melt mixing + 3D printing		-	57	-	50	2020	[83]
					-		-	150		
					-		-	33		
					-		-	120		

EUG—*Eucommia ulmoides* gum; EVA—poly(ethylene-co-vinyl acetate); NR—natural rubber; CW—carnauba wax; PEO—polyolefin elastomer; PW—paraffin wax; PBS—Poly(butylene succinate); PCL—poly(ϵ-caprolactone); PCL-Py—pyrene-containing poly(ϵ-caprolactone); PLA—poly(lactic) acid; POE—poly(ethylene-co-octene); TPU—Thermoplastic polyurethane; fMWCNT—functionalized MWCNT; hMWCNT—hybrid MWCNT.

**Table 9:** SMP/CNT with self-healing ability, their polymer matrix, filler, trigger to initiate self-healing, and mechanism through which self-healing is enabled.

Polymer	Trigger	Filler	Mechanism	Reference
fu-PCL	Electrical	MWCNT	rDA	[216]
fu-PK				[217]
TPU/SBS			Reversible C−ON bonds	[218]
PLA/PCL		hCNT	SME	[72]
ER	Light	MWCNT	Reversible disulfide bonds	[193]
fu-SBS			rDA	[219]
mPE-g-AA			SME	[109]
PPC				[220]
TPU/PCL				[199]
PVA		hMWCNT		[221]
		fCNT		[222]
EVA		hMWCNT		[106]

fu-PCL—furan-modified poly(ϵ-caprolactone); fu-PK—furan modified polyketone; PLA—poly(lactic) acid; PCL—poly(ϵ-caprolactone); TPU—Thermoplastic polyurethane; SBS—poly(styrene-butadiene-β-styrene); ER—epoxy resin; EVA—poly(ethylene-co-vinyl acetate); fu-SBS—furan-modified poly(styrene-butadiene-β-styrene); mPE-g-AA—Acrylic acid-functionalized metallocene polyethylene; PPC—poly(propylene) carbonate; hCNT—hybrid CNT; hMWCNT—hybrid MWCNT; fCNT—functionalized CNT.

## Abbreviations

The following abbreviations are used in this manuscript:

ABS	Acrylonitrile butadiene styrene
AFM	Atomic Force Microscopy
AR	acrylic resin
BR	benzoxazine resin
CAD	computer-aided design
CB	carbon black
CE	cyanate ester
CF	carbon fibers
CNSL	Cashew nut shell liquid
CNT	carbon nanotubes
CTE	coefficient of thermal expansion
CW	carnauba wax
DA	dopamine
DLP	digital light process
EM	electromagnetic
EOC	Ethylene-α-octene copolymer
EPAc	Epoxy Acrylate
ER	epoxy resin
ESO	Epoxidized soybean oil
et-SMP	electrothermal shape-memory polymer
EUG	*Eucommia ulmoides* gum
EVA	poly(ethylene-co-vinyl acetate)
fCNT	functionalized carbon nanotubes
FDM	fused deposition modeling
fMWCNT	functionalized multi-walled carbon nanotubes
fu	furan-grafted
Gr	graphene
HBPU	Hyperbranched Polyurethane
hCNT	hybrid carbon nanotubes
hMWCNT	hybrid multi-walled carbon nanotubes
hSWCNT	hybrid single-walled carbon nanotubes
IR	infra-red
LCE	liquid crystal elastomer
LDPE	low-density polyethylene
mPE-g-AA	Acrylic acid-functionalized metallocene polyethylene
MW	Microwave
MWCNT	multi-walled carbon nanotubes
NIR	near infra-red
NR	natural rubber
P(AM-co-OA)	poly(acrylamide-octadecyl acrylate)
PAEK	polyaryletherketone
PANI	polyaniline
PBS	Poly(butylene succinate)
PCL	poly(ϵ-caprolactone)
PCL-Py	pyrene-containing poly(ϵ-caprolactone)
PCO	Polycyclooctene
PE	Polyethylene
PEG	Polyethylene glycol
PEGDMA	Polyethylene glycol dimethacrylate
PEO	polyolefin elastomer
PEU	Polyester urethane
PK	polyketone
PLA	poly(lactic) acid
PNIPAAm	Poly(N-isopropylacrylamide)
POE	poly(ethylene-co-octene)
POP	palm oil polyol
PPC	poly(propylene) carbonate
PS	polystyrene
pt-SMP	photothermal shape-memory polymer
PU	thermoset polyurethane
PVA	polyvinyl alcohol
PVC	Polyvinyl Chloride
PVDF	polyvinylidene fluoride
PW	paraffin wax
$R_{f}$	shape fixity ratio
$R_{r}$	shape recovery ratio
rDA	retro Diels–Alder
RF	radio frequency
rGO	reduced graphene oxide
RPSM	Reversible plasticity shape-memory
SBS	poly(styrene-β-butadiene-β-styrene)
SIS	poly(styrene-β-isoprene-β-styrene)
SLA	Stereolithography
SME	shape-memory effect
SMP	shape-memory polymers
SWCNT	single-walled carbon nanotubes
$T_{g}$	glass transition temperature
$T_{m}$	melting temperature
$T_{trans}$	transition temperature
t-SMP	thermally responsive shape-memory polymer
TC	thermal conductivity
TPI	Trans-1,4-polyisoprene
TPU	thermoplastic polyurethane
UHMWPE	Ultra-High-Molecular-Weight Polyethylene
UV	ultra-violet
Vis	Visible
